# Differential Metabolic and Transcriptional Responses of Gilthead Seabream (*Sparus aurata*) Administered with Cortisol or Cortisol-BSA

**DOI:** 10.3390/ani11113310

**Published:** 2021-11-19

**Authors:** Jorge Aedo, Daniela Aravena-Canales, Ignacio Ruiz-Jarabo, Ricardo Oyarzún, Alfredo Molina, Gonzalo Martínez-Rodríguez, Juan Antonio Valdés, Juan Miguel Mancera

**Affiliations:** 1 Department of Biological Sciences, Faculty of Life Sciences, Andres Bello University, Santiago 8320000, Chile; jor.aedo@uandresbello.edu (J.A.); da.aravena@uandresbello.edu (D.A.-C.); amolina@unab.cl (A.M.); 2 Interdisciplinary Center for Aquaculture Research (INCAR), Concepción 4030000, Chile; 3 Department of Biology, Faculty of Marine and Environmental Sciences, Instituto Universitario de Investigación Marina (INMAR), Campus de Excelencia Internacional del Mar (CEI-MAR), University of Cádiz, 11519 Puerto Real, Spain; ignaru02@ucm.es (I.R.-J.); juanmiguel.mancera@uca.es (J.M.M.); 4 Department of Animal Physiology, Faculty of Biology, University Complutense of Madrid, 28040 Madrid, Spain; 5 Institute of Marine and Limnological Sciences, Faculty of Sciences, University Austral of Chile, Valdivia 5110652, Chile; r.oyarzun.salazar@gmail.com; 6 Department of Marine Biology and Aquaculture, Instituto de Ciencias Marinas de Andalucía (ICMAN-CSIC), 11519 Puerto Real, Spain; gonzalo.martinez@csic.es

**Keywords:** cortisol, energetic metabolism, fish, glucocorticoids, *Sparus aurata*, stress response

## Abstract

**Simple Summary:**

Cortisol is a key stress hormone in teleosts. Cortisol exerts its effects through genomic—and membrane-initiated mechanisms, however, the role of the latter in long-term stress responses is unknown. Here, we treated *Sparus aurata* with cortisol or cortisol-BSA (exclusive inductor to membrane-initiated effects) to emulate a long-term stress situation. We found that cortisol, but not cortisol-BSA, promotes energy substrate mobilization in the liver, together with the regulation of metabolism-related genes. We suggest that genomic cortisol actions exclusively participate in metabolic responses during prolonged treatment using cortisol in *S. aurata*. This study contributes to the current knowledge on cortisol’s involvement in stress responses in fish.

**Abstract:**

Cortisol is the main glucocorticoid hormone promoting compensatory metabolic responses of stress in teleosts. This hormone acts through genomic and membrane-initiated actions to exert its functions inside the cell. Experimental approaches, using exogenous cortisol administration, confirm the role of this hormone during short (minutes to hours)- and long-term (days to weeks) responses to stress. The role of membrane-initiated cortisol signaling during long-term responses has been recently explored. In this study, *Sparus aurata* were intraperitoneally injected with coconut oil alone or coconut oil containing cortisol, cortisol-BSA, or BSA. After 3 days of treatment, plasma, liver, and skeletal muscle were extracted. Plasma cortisol, as well as metabolic indicators in the plasma and tissues collected, and metabolism-related gene expression, were measured. Our results showed that artificially increased plasma cortisol levels in *S. aurata* enhanced plasma glucose and triacylglycerols values as well as hepatic substrate energy mobilization. Additionally, cortisol stimulated hepatic carbohydrates metabolism, as seen by the increased expression of metabolism-related genes. All of these responses, observed in cortisol-administered fish, were not detected by replicating the same protocol and instead using cortisol-BSA, which exclusively induces membrane-initiated effects. Therefore, we suggest that after three days of cortisol administration, only genomic actions are involved in the metabolic responses in *S. aurata*.

## 1. Introduction

In recent decades, efforts have been made in aquaculture to improve management practices and the monitoring of animal welfare trough the evaluation of novel and/or classical stress indicators [1,2,3,4]. Cortisol is the main hormone that promotes compensatory metabolic response to stress in teleost fish [5,6]. Stressful events in natural and farming environments trigger plasma cortisol enhancement, which latently promotes the energetic substrate mobilization in liver and skeletal muscles, allowing for fish acclimatization and homeostasis recovery [7,8]. The relevance of cortisol as a key mediator of the stress response in fish has been extensively studied, including through in vivo experiments associated with exogenous hormone administration using saline and/or oil vehicles [9,10,11]. In gilthead seabream (*Sparus aurata*) long-term cortisol administration, using diet or slow-release implants, enhanced catabolism together with the regulation of several gluconeogenesis, glycogenolysis and proteolysis related genes [12,13,14].

Overall, cortisol metabolic effects are associated with classical/genomic mechanisms involving interaction of intracellular glucocorticoid (GR) and mineralocorticoid receptors (MR), and the subsequent regulation of target genes [15]. Additionally, cortisol can also interact with plasma membrane-localized components activating rapid/intracellular GR-independent signaling pathways with novel features related to early compensatory responses to stress [16,17,18]. In this context, we recently determined that short-term cortisol treatment (1 to 6 h) triggers a metabolic response in the liver of gilthead seabream, characterized by glucose and lactate plasma levels enhancement, hepatic glucose, and glycogen mobilization, as well as the regulation of both glycolysis and gluconeogenesis-related genes [16]. All these metabolic effects were further validated using a specific impermeable-membrane cortisol analog, cortisol-BSA [16,17]. Nevertheless, as of yet, the contribution of long-term membrane-initiated cortisol action on metabolic responses in fish remains unknown.

In this work, we evaluate the potential contribution of the prolonged effects of cortisol (three days of treatment) on metabolic and transcriptional responses of gilthead seabream by using cortisol or cortisol-BSA dissolved in coconut oil implanted intraperitoneally. For this purpose, *S. aurata* juveniles were treated with cortisol or with the membrane impermeable cortisol analog, cortisol-BSA, dissolved in coconut oil. After 72 h, plasma cortisol values and several metabolites in the plasma, liver, and skeletal muscle were determined. In addition, metabolic-related gene expression was also assessed at the hepatic and skeletal muscle levels. The results were discussed in relation to the possible contribution of membrane-initiated cortisol actions on the regulation of metabolic and transcriptional responses of *S. aurata*.

## 2. Materials and Methods

### 2.1. Experimental Design

Immature gilthead seabream (*S. aurata*) (24.74 ± 0.29 g body mass, mean ± SEM, *n* = 32) were provided by Servicios Centrales de Investigación en Cultivos Marinos (SCI-CM, CASEM, University of Cadiz, Puerto Real, Cádiz, Spain; Spanish Operational Code REGA ES11028000312). Fish were randomly distributed in eight 100-L tanks (~2.5 kg/m^−3^ density) and kept under a natural photoperiod (12:12 h LD) (march, 2018), constant temperature (18 °C) in a flow-through system. Fish were fed by hand twice per day (9:30 and 15:30 h; 2% of tank biomass per day) with commercial pellets. After ten days of acclimation, fish were anesthetized with 2-phenoxyethanol (0.3 mL/L) and treated with the following intraperitoneal implants (10 μL/g body weight): (i) coconut oil alone (Sigma-Aldrich, San Luis, MO, USA) (sham group), (ii) coconut oil containing cortisol (0.138 µmol per g of fish) (Sigma-Aldrich) (cortisol group), (iii) coconut oil containing cortisol-BSA (0.138 µmol per g of fish) (US biological, USA) (cortisol-BSA group), or iv) coconut oil containing BSA alone (0.001784 mg per g of fish) (Sigma-Aldrich) (BSA group). This latter group was included to consider the potential effects of BSA within the cortisol-BSA complex. The experiment was performed using duplicate tanks for each group and fish were not fed during the experiment.

After 72 h of treatment, all fish were euthanized through an overdose of 2-phenoxyethanol (1 mL/L) and sampled. Plasma was obtained by centrifugation of the blood (3 min, 10,000× *g*, 4 °C), snap frozen in liquid nitrogen and stored at −80 °C until further analysis. Liver and skeletal muscle were excised, and the biopsies collected in microtubes were snap frozen in liquid nitrogen and stored at −80 °C until the assay of metabolites. Additionally, other liver and skeletal muscle samples were collected and placed into tubes with 10-volumes (*v*/*w*) of RNA*later*™ Soln. (Invitrogen by Thermo Fisher Scientific, Waltham, MA, USA), held for 24 h at 4 °C and stored at −20 °C until total RNA isolation.

### 2.2. Measurement of Plasma Cortisol and Tissue Metabolites

Plasma cortisol levels were measured by EIA kit (Arbor assays), which was previously validated in *S. aurata* [16,19]. Plasma glucose, lactate, and triacylglycerols (TAG) were quantified with the following Spinreact kits (Barcelona, Spain): HK Ref. 1001200, Lactate Ref. 1001330, and TAG Ref: 41030, respectively, adapted to 96-well microplates. Protein plasma levels were measure using a Pierce BCA Protein Assay Kit (Thermo Scientific, Hanover Park, IL, USA).

For the analysis of tissue metabolites, skeletal muscle and liver were finely minced on an ice-cooled Petri dish and were homogenized by ultrasonic disruption in 7.5 volumes of ice-cold 0.6 N perchloric acid, neutralized using 1 M KCO_3_, centrifuged (30 min, 3220× *g* and 4 °C), after which the supernatant was stored until used for metabolites determination. Tissue lactate and triacylglycerols levels were determined spectrophotometrically with commercial kits (Spinreact, see before). The tissue glycogen concentration was assessed as described by Keppler and Decker [20], whereby tissue homogenates are incubated for 2 h at 37 °C with and without amyloglucosidase (Sigma-Aldrich A7420, San Luis, MO, USA) to break down glycogen molecules into glucose. The total glucose in both incubations was determined with a commercial kit (Spinreact, see above), and the glycogen content was expressed as glucose equivalents after free glucose subtraction [16,21].

### 2.3. RNA Extraction and cDNA Synthesis

The total RNA of the *S. aurata* liver and skeletal muscle were obtained using the NucleoSpin RNA II kit (Macherey-Nagel) in accordance with the manufacturer’s instructions. The gDNA elimination step was performed using on-column RNase-free DNase digestion, following the manufacturer’s instructions. The RNA integrity was evaluated using 2100 Bioanalyzer using the RNA 6000 Nano Kit (Agilent Technologies, Santa Clara, CA, USA) and RNA quantification was estimated using Qubit^®^ 2.0 Fluorometer (Life Technology, Carlsbad, CA, USA). Only RNA with RIN > 8.0 was used for cDNA synthesis. Retro transcription was carried out with the qScriptTM cDNA Synthesis Kit (Quanta BioSciences), using 500 ng from liver and skeletal muscle RNA as an input, following the manufacturer’s instructions.

### 2.4. Real Time-PCR

Real time PCR were performed using the Biorad qPCR system (Quanta BioSciences, Gaithersburg, MD, USA) in a final volume of 20 µL. Details of the qPCR reaction mixture are indicated in [16]. Several calibration plots with serial dilutions of input total RNA from liver and skeletal muscle had amplification efficiencies between 90.3–105.7%, and 95.1–103.8%, respectively.

The PCR profile was as follows: 95 °C, 10 min; [95 °C, 20 s; 60 °C, 30 s] × 40 cycles; melting curve [60 °C to 95 °C, 20 min], 95 °C, 15 s. The results were normalized against beta actin (*actb*) and elongation factor 1a (*ef1a*) as housekeeping genes. Relative gene quantification was performed using the ΔΔCT method [22]. Candidate sequences corresponding to aldolase (*aldo*), phosphoenolpyruvate carboxykinase (*pepck*), glucose 6 phosphatase (*g6pc*), *actb* and *ef1α* were available in the NCBI database. Conversely, a sequence of phosphoglycerate mutase 1 (*pgam1*), enolase 3 (*eno3*), atrogin-1, and muscle RING-finger protein-1 (*murf-1*) was obtained from the available database belonging to the *S. aurata* sequencing project [23]. Primers were designed using the Primer 3 available tool (http://frodo.wi.mit.edu/primer3/; accessed on date 3 February 2018) and validated in NetPrimer (http://www.premierbiosoft.com/netprimer/; accessed on date 6 February 2018) and Oligo analyzer 3.1 (https://www.idtdna.com/calc/analyzer/; accessed on date 6 February 2018) available online tools. Finally, the primers sequence of the glucocorticoid receptor 1 (*gr1*), glucocorticoid receptor 2 (*gr2*) and mineralocorticoid (*mr*) were obtained from [24].

### 2.5. Statistical Analysis

The normality and homogeneity of variances were analyzed using the Kolmogorov-Smirnov’s and the Levene’s tests, respectively. All data were analyzed using a one-way ANOVA with treatment as the factor of variance, followed by a post-hoc Tukey’s honestly significant difference (HSD) test. All statistical analysis were performed using the Graph Prism 7.0 software (GraphPad Software, Inc., San Diego, CA, USA). Finally, differences were considered using a *p* value of <0.05.

## 3. Results

### 3.1. Plasma Indicators in S. aurata Administered with Cortisol or Cortisol-BSA

**Figure 1:**
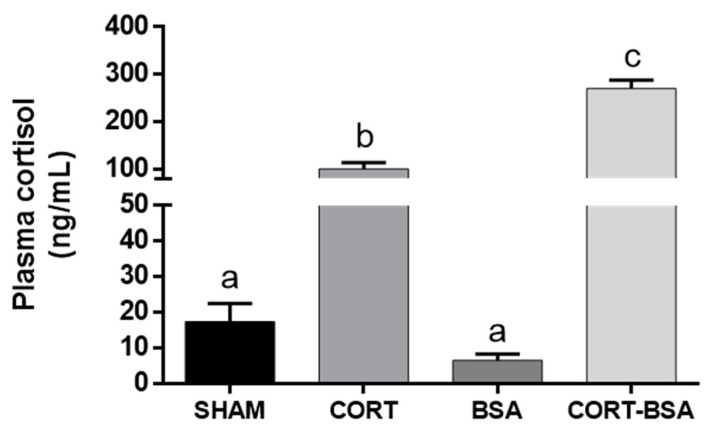
Plasma cortisol levels in vehicle (sham-control), cortisol, BSA and cortisol-BSA administered fish at 72 h post-treatment. Results are expressed as mean ± SEM (*n* = 8). Different letters indicate significant differences between groups (*p* value < 0.05). All data were analyzed using one-way ANOVA and Tukey’s HSD as a post-hoc test.

Plasma cortisol levels significantly increased in the cortisol (100.37 ± 13.12 ng/mL) and cortisol-BSA (270.67 ± 17.17 ng/mL) injected groups compared to the sham (17.41 ± 5.13 ng/mL) and BSA (6.52 ± 1.75 ng/mL) groups, respectively (Figure 1).

The increase of cortisol resulting from the administration of cortisol, but not cortisol-BSA, enhanced glucose (6.95 ± 0.39 mmol/L) and TAG (257.39 ± 24.64 mg/dL) plasma levels compared to the vehicle group (glucose: 4.07 ± 0.19 mmol/L and TAG: 92.64 ± 14.12 mg/dL) (Table 1). Finally, neither plasma proteins nor lactate displayed significant differences between the cortisol or cortisol-BSA groups against vehicle or BSA groups (Table 1).

### 3.2. Energetic Metabolites in Liver and Skeletal Muscle of S. aurata Administered with Cortisol or Cortisol-BSA

With respect to liver energetic metabolites, the glycogen content increased in the cortisol group (0.79 ± 0.09 mg/g) but not in the cortisol-BSA administered group (0.23 ± 0.06 mg/g) (Table 2).

According to the analyzed skeletal muscle energy metabolites, we only observed a non-significant tendency of increase in TAG for the cortisol-treated group (2.94 ± 1.17 mg/g) but not in the cortisol-BSA administered group (0.46 ± 0.15 mg/g) compared to the vehicle group (1.30 ± 0.55 mg/g) (Table 3). Finally, there were no changes in skeletal muscle glucose, glycogen, and lactate of *S. aurata* administered with cortisol or cortisol-BSA (Table 3).

### 3.3. Corticosteroid Receptor-Related Genes Expression in Liver and Skeletal Muscle of S. aurata Administered with Cortisol or Cortisol-BSA

**Figure 2:**
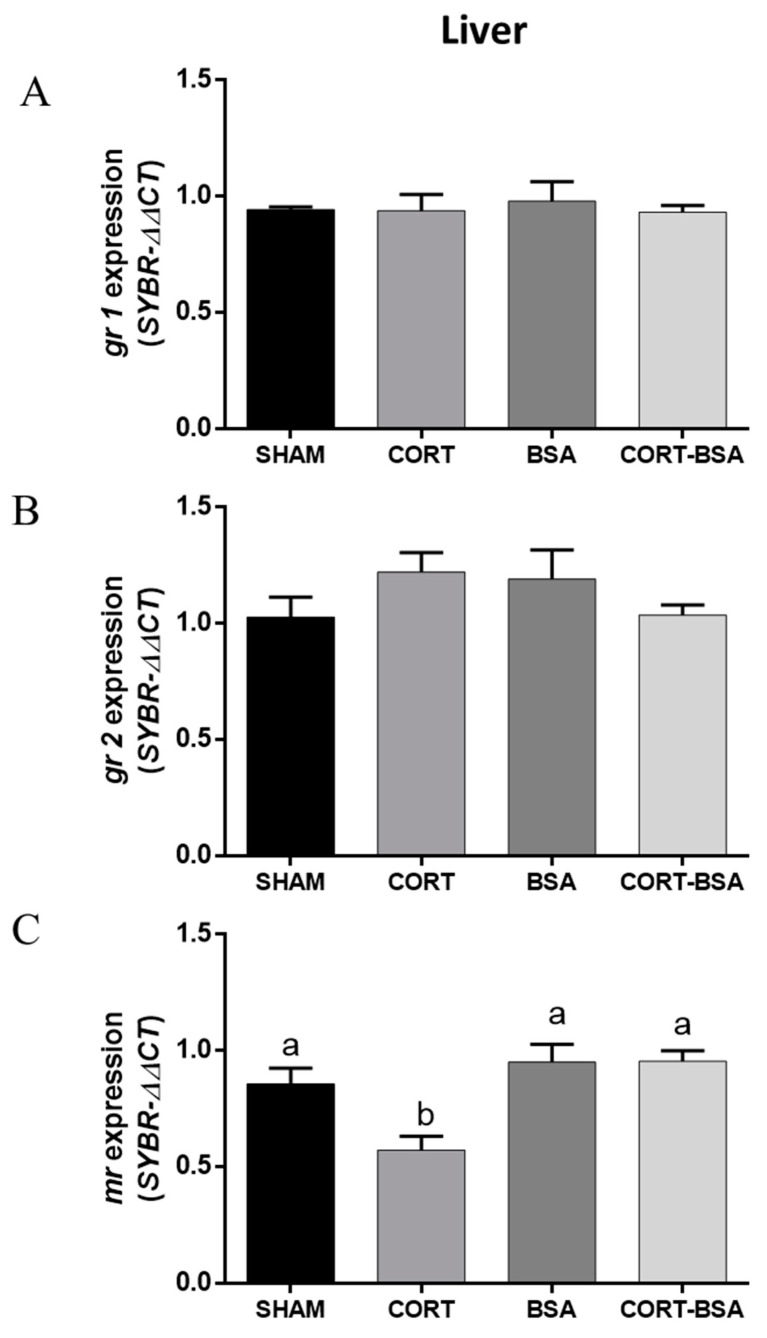
Corticosteroid-receptors gene expression in liver. Results of *gr1* (**A**), *gr2* (**B**) and *mr* (**C**) expression in vehicle (sham-control), cortisol, BSA and cortisol-BSA administered fish at 72 h post-treatment. Results are expressed as mean ± SEM (*n* = 8). Different letters indicate significant differences between groups (*p* value < 0.05). All data were analyzed using one-way ANOVA and Tukey’s HSD as a post hoc test.

By analyzing corticosteroid receptor-related gene expression, it was found that *gr1* and *gr2* mRNA levels in liver did not change between the cortisol or cortisol-BSA groups compared to in the vehicle group (Figure 2A,B). However, *mr* mRNA levels decreased in cortisol (0.57 ± 0.06 relative expression) but not for cortisol-BSA (0.95 ± 0.08 relative expression) administered fish compared to the vehicle group (0.95 ± 0.05 relative expression) (Figure 2C).

**Figure 3:**
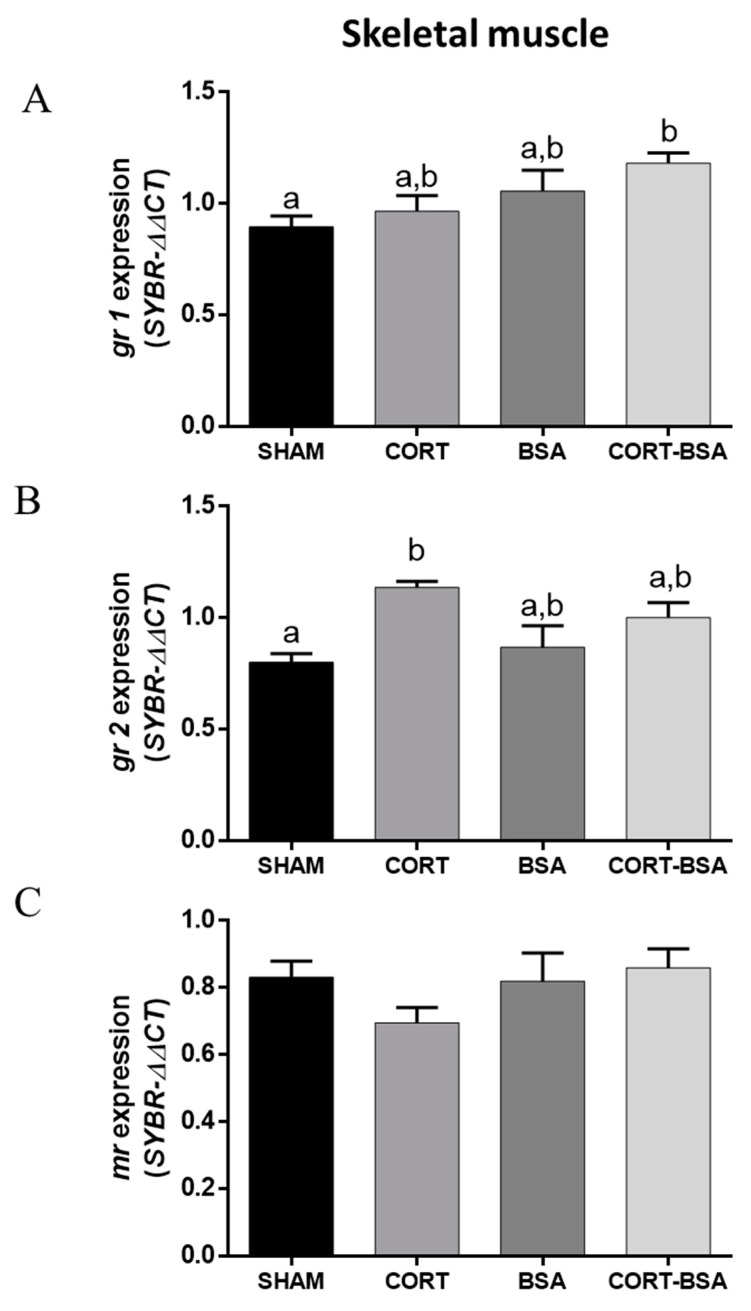
Corticosteroid-receptors gene expression in skeletal muscle. Results of *gr1* (**A**), *gr2* (**B**) and *mr* (**C**) expression in vehicle (sham-control), cortisol, BSA and cortisol-BSA administered fish at 72 h post-treatment. Results are expressed as mean ± SEM (*n* = 8). Different letters indicate significant differences between groups (*p* value < 0.05). All data were analyzed using one-way ANOVA and Tukey’s HSD as a post-hoc test.

On other hand, there were no changes observed for *gr1* and *mr* expression in the skeletal muscle of the *S. aurata* administered with cortisol or cortisol-BSA (Figure 3A–C). However, *gr2* mRNA levels increased in cortisol but not in cortisol-BSA injected fish (Figure 3B).

### 3.4. Glucose Metabolism—And Atrophy-Related Genes in S. aurata Administered with Cortisol or Cortisol-BSA

**Figure 4:**
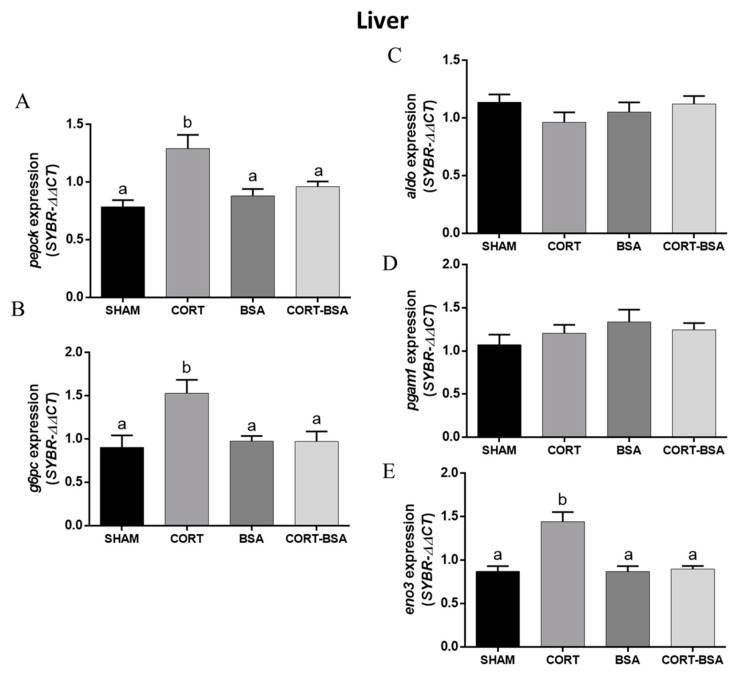
Glucose-metabolism related genes expression in liver. Expression of gluconeogenesis-related genes *pepck* (**A**) and *g6pc* (**B**), and glycolysis-related genes *aldo* (**C**), *pgam1* (**D**), *eno3* (**E**) were evaluated in vehicle (sham-control), cortisol, BSA and cortisol-BSA administered fish at 72 h post-treatment. Results are expressed as mean ± SEM (*n* = 8). Different letters indicate significant differences between groups (*p* value < 0.05). All data were analyzed using one-way ANOVA and Tukey’s HSD as a post hoc test.

The gluconeogenesis-related genes *pepck* and *g6pc* had an enhanced expression in the liver as a result of cortisol administration, but not for cortisol-BSA (Figure 4A,B). Additionally, we found a high correlation between both hepatic gluconeogenesis-related genes and plasma glucose levels (Supplementary Figure S1). With regard to glycolysis-related gene expression, *eno3* increased its expression in liver for cortisol but not cortisol-BSA administered *S. aurata* (Figure 4E). On the other hand, neither hepatic *aldol* nor *pgam1* expression is found to be modulated by cortisol or cortisol-BSA administration (Figure 4C,D).

**Figure 5:**
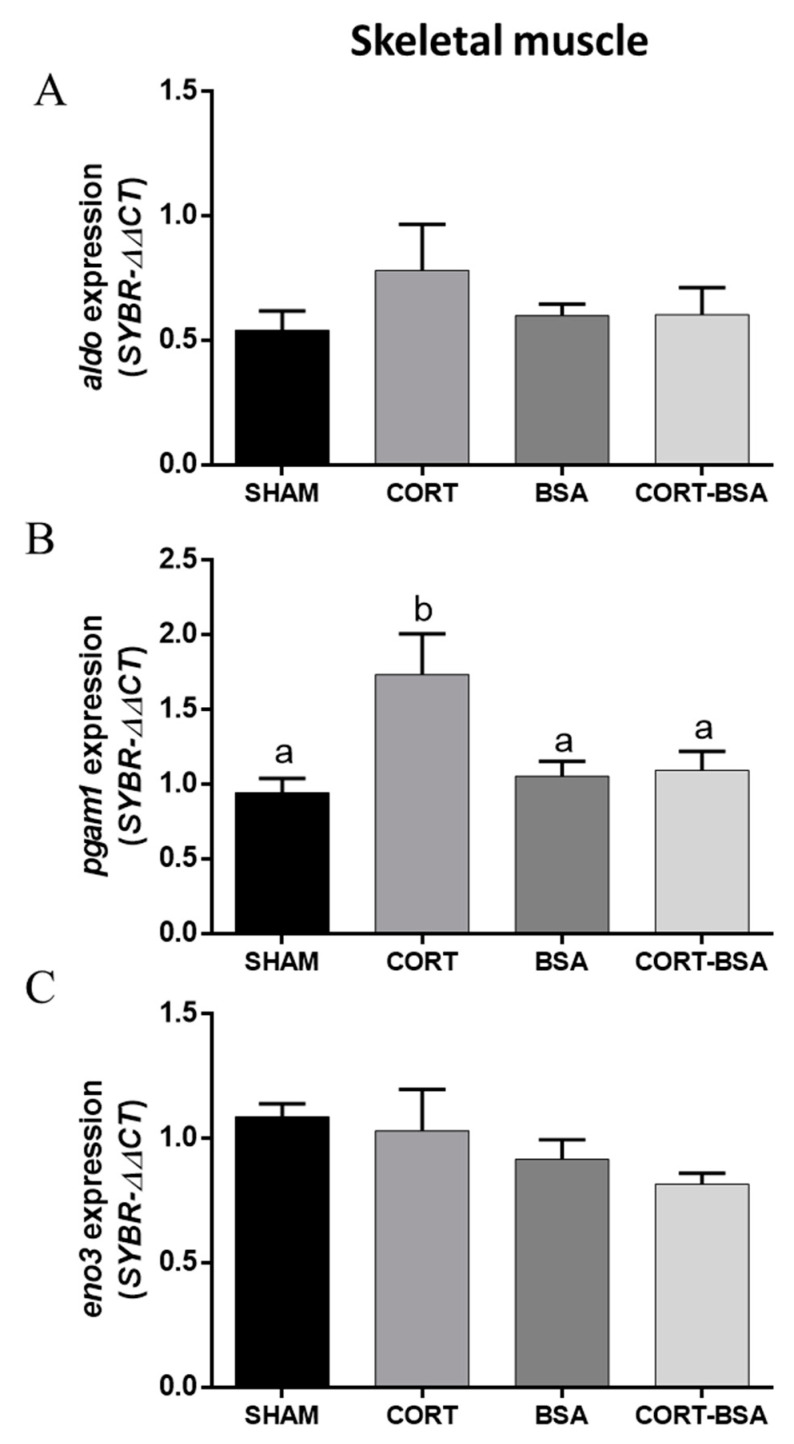
Glucose-metabolism related genes expression in skeletal muscle. Expression of glycolysis-related genes *aldo* (**A**), *pgam1* (**B**), *eno3* (**C**) were evaluated in vehicle (sham-control), cortisol, BSA and cortisol-BSA administered fish at 72 h post-treatment. Results are expressed as mean ± SEM (*n* = 8). Different letters indicate significant differences between groups (*p* value < 0.05). All data were analyzed using one-way ANOVA and Tukey’s HSD as a post-hoc test.

Otherwise, the glycolysis-related gene *pgam1* increased its expression (0.74 ± 0.27 relative expression) in the skeletal muscle under cortisol but not cortisol-BSA treatment (Figure 5B), while there were no changes in the expression of *eno3* or *aldo* in this tissue (Figure 5A–C).

**Figure 6:**
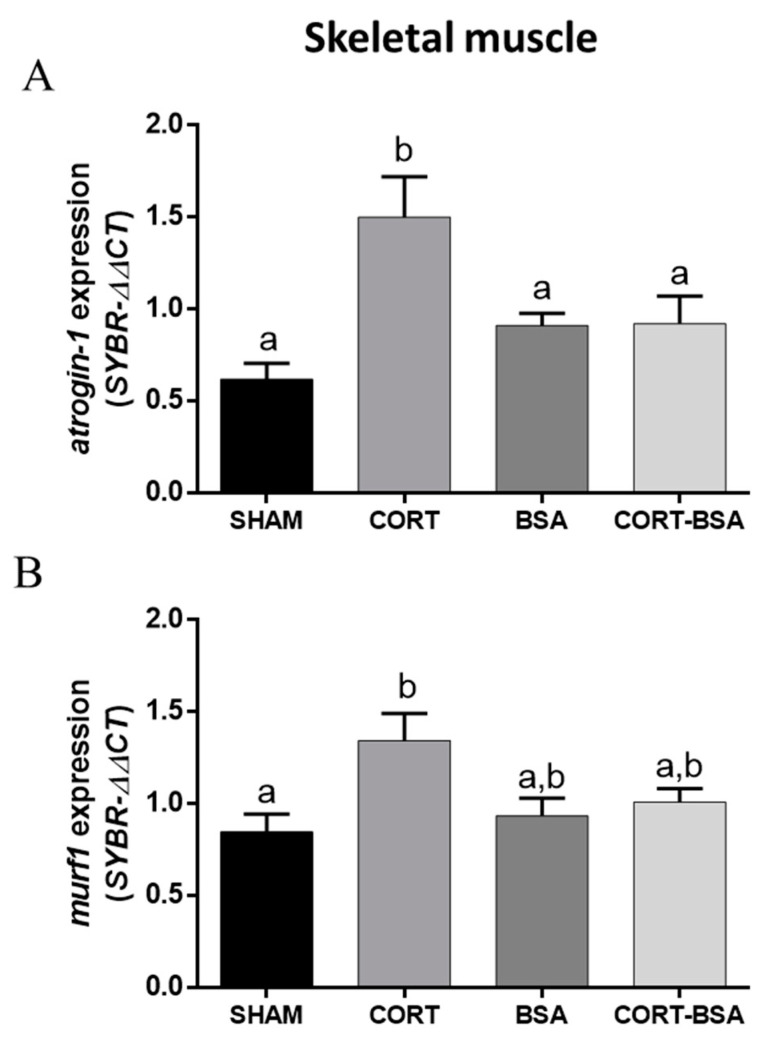
Skeletal muscle atrophy-related genes expression. Expression of *atrogin-1* (**A**) and *murf1* (**B**) were evaluated in vehicle (sham-control), cortisol, BSA and cortisol-BSA administered fish at 72 h post-treatment. Results are expressed as mean ± SEM (*n* = 8). Different letters indicate significant differences between groups (*p* value < 0.05). All data were analyzed using one-way ANOVA and Tukey’s HSD as a post-hoc test.

Finally, regarding the atrophy-related genes expression in skeletal muscle of *S. aurata* administered with cortisol or cortisol-BSA, *atrogin-1* and *murf1* had an enhanced expression (1.50 ± 0.22 and 1.34 ± 0.15 relative expression, respectively) the in cortisol administered group, but not in the cortisol-BSA administered group, compared to the vehicle group (0.62 ± 0.09 and 0.85 ± 0.10 relative expression, respectively) (Figure 6A,B).

## 4. Discussion

Cortisol is an essential glucocorticoid involved in the regulation of physiological and metabolic responses of teleost fish under basal and stressful conditions [5,12,25,26]. Cortisol-related effects on metabolism, growth, immunity, and other physiological processes have been evaluated through the exogenous administration of cortisol (in saline or oil as a vehicle) to determine its potential effects under acute or chronic stress [27,28,29]. Due to cortisol’s ability to activate intracellular and extracellular initiated actions, the use of a plasma membrane impermeable analog of cortisol, such as cortisol-BSA, seems useful to discriminate membrane-initiated cortisol effects during long-term stress responses [16,17,18]. In the present work, we found that, in *S. aurata* plasma and tissue, energy metabolites as well as hepatic and skeletal muscle expression of selected metabolism-related genes are mediated by cortisol but not cortisol-BSA treatment. We previously determined that cortisol-BSA is suitable to induce rapid specific membrane-initiated effects (1-6 h) in *S. aurata* [16], in agreement with the reports for other teleosts [30,31]. Additionally, the inability to cross the plasma membrane and its stability over an extended period has been proved in steroids attached to BSA molecules [6,17,32].

Particularly, we determined that cortisol, but not cortisol-BSA, increased the hepatic glycogen content, which has been previously reported in *S. aurata* juveniles injected with slow-release cortisol implants [33]. Furthermore, the plasma cortisol levels that were reached in our in vivo experiment are similar to those observed in confined *S. aurata* for 24 to 72 h [34,35]. In addition, cortisol-administered fish enhanced plasma TAG values, similar to the levels found in this species during the recovery period after a stressful process [36]. Since cortisol-mediated stress has been linked to the regulation of the lipid metabolism, we suggest that genomic cortisol actions specifically mediate this response [33,37]. Taking into account these results, and the absence of metabolism-related processes regulated by cortisol-BSA, we postulate that metabolic reprogramming in *S. aurata* after three days of cortisol-mediated stress could be triggered exclusively by intracellular GR signaling. This is in agreement with previous studies conducted for other teleost fish species [38,39].

Cortisol is capable of interacting with glucocorticoid (GR) and mineralocorticoid (MR) receptors to exert its effects in the cell [15]. Most fish, including *S. aurata*, demonstrate two or more GR with different expression patterns under stress/cortisol treatment [25,40,41]. We found that hepatic *gr1* and *gr2* mRNA levels remained unaltered after cortisol or cortisol-BSA treatments. Our results are therefore in agreement with previous studies that reported no variation in *gr1* or *gr2* expression levels in rainbow trout after five days of cortisol treatment, coinciding with the lack of variation in *gr* mRNA levels in *S. aurata* after seven days of cortisol treatment [13,41]. On the other hand, even though fish do not synthesize aldosterone (a mineralocorticoid), which represents the canonical ligand of MR, fish do express MR in different tissues including liver and skeletal muscle [16,42]. We found that cortisol, but not cortisol-BSA, decreased *mr* hepatic expression in *S. aurata*. Interestingly, similar approaches using slow-release cortisol implants in *Cyprinus carpio* induced an opposite behavior in this expression [43]. We speculate on the presence and differentiated control of species-specific *mr* expressed in teleosts. However, the role of MR signaling pathways in the liver of fish is poorly understood and requires further study [42]. Regarding the skeletal muscle of *S. aurata*, cortisol, but not cortisol-BSA, treatment enhanced *gr2* expression levels after three days. This agrees with the reported results in the fine flounder (*Paralichthys adspersus*) for which 28 days of chronic stress increased plasma cortisol levels alongside *gr2*, but not *gr1*, mRNA levels in the skeletal muscle [44]. Additionally, *gr1* and *gr2* show differentiated expression patterns during an acute stress response in *S. aurata* [24]. Therefore, we postulate that GR2 regulates the cortisol effects in the skeletal muscle of this species. It is important to note that *gr1* expression increased in in cortisol-BSA group compared to the sham-control group, but not in the BSA group. In this context we cannot rule out whether BSA or membrane-initiated cortisol actions mediate this effect. We also observed that both *atrogin-1* and *murf1* are regulated by cortisol but not by cortisol-BSA in this tissue. Both are critical atrophy-related genes that increase in expression under stress and/or glucocorticoid stimulus in fish [45,46]. Moreover, *S. aurata* subjected to stress-related conditions, such as fasting or exhaustive swimming, demonstrated an increased *atrogin-1* and *murf1* expression in the skeletal muscle [46,47]. We have recently reported the contribution of membrane-initiated cortisol actions in the early regulation of both atrogenes in isolated fish myotubes [48]. However, after prolonged cortisol treatment, atrophy-related processes are mediated by genomic cortisol signaling. This agrees with the reported muscular proteolytic action of cortisol as well as with its negative influence on growth during chronic stress situations [49,50]. 

It is well known that cortisol-mediated stress induces changes in the hepatic expression of gluconeogenesis-related genes in fish [13,51,52]. The observed hepatic *pepck* and *g6pc* expression enhancement in *S. aurata* administered with cortisol agree with this idea. The fact that cortisol-BSA does not modulate the expression of *pepck* and *g6pc* after 3 days of treatment leads us to hypothesize that both key gluconeogenesis-related genes are exclusive targets of cortisol action in teleosts. In particular, *pepck* presents a glucocorticoid response element (GRE), supporting the idea of its direct regulation by cortisol during the stress response [38]. On other hand, we have previously shown that *S. aurata* hepatic *g6pc*, but not *pepck*, expression is mediated by cortisol-BSA during early treatment (1 to 6 h) [16]. Overall, these data suggest that membrane-initiated cortisol actions participate in early-term gluconeogenesis regulation through *g6pc* induction, followed by cortisol genomic long-term actions trough the increase of both *pepck* and *g6pc* expression. Regarding those genes related to the glycolysis-metabolism via cortisol, the level of hepatic *eno3* and skeletal muscle *pgam1* expression enhancements are in agreement with previous reports in teleosts, including *S. aurata*, that are subjected to different stressors [53,54]. In this context, glycolysis, exclusively mediated by genomic cortisol actions, is critical for releasing energy in fish so as to overcome stress [5,6,25]. Finally, it is important to mention that all metabolic and transcriptional effects mediated by cortisol were observed using one experimental period (3 days). In this context, the potential effects of membrane or genomic-initiated cortisol actions during more prolonged treatments (weeks to months) presents a potential research avenue for future studies. 

## 5. Conclusions

Considering all of the metabolic and transcriptional results, we propose that a rapid cortisol signaling pathway plays a role in gluconeogenesis-glycolysis and atrophy-related processes. Its effects are evident at an early stage during the stress response in *S. aurata*, and is exclusively followed by the regulation of genomic cortisol pathways. Overall, our results suggest that genomic cortisol actions exclusively (potentially mediated by intracellular GR) participate in metabolic responses during prolonged treatment (3 days), as was observed in our study, using cortisol in *S. aurata*.

## Figures and Tables

**Table 1:** Plasma glucose, triacylglycerols, lactate and proteins levels in vehicle (sham-control), cortisol, BSA and cortisol-BSA administered fish at 72 h post-treatment. Results are expressed as mean ± SEM (*n* = 8). Different letters indicate significant differences between groups (*p* value < 0.05). All data were analyzed using one-way ANOVA and Tukey’s HSD as a post-hoc test.

Parameter	SHAM	CORT	BSA	CORT-BSA
Glucose (mmol/L)	4.07 ± 0.19 ^a^	6.95 ± 0.39 ^b^	4.07 ± 0.25 ^a^	3.76 ± 0.16 ^a^
Triacylglycerols (mg/dL)	92.64 ± 14.12 ^a^	257.39 ± 24.64 ^b^	66.69 ± 2.76 ^a^	64.52 ± 6.69 ^a^
Lactate (mmol/L)	2.11 ± 0.28	2.58 ± 0.24	1.60 ± 0.26	1.66 ± 0.23
Protein (mg/mL)	31.76 ± 1.27	34.76 ± 1.16	28.64 ± 2.11	30.39 ± 0.91

**Table 2:** Hepatic glucose, triacylglycerols, lactate and glycogen content in vehicle (sham-control), cortisol, BSA and cortisol-BSA administered fish at 72 h post-treatment. Results are expressed as mean ± SEM (*n* = 8). Different letters indicate significant differences between groups (*p* value < 0.05). All data were analyzed using one-way ANOVA and Tukey’s HSD as a post hoc test.

Parameter	SHAM	CORT	BSA	CORT-BSA
Glucose (mg/g)	1.05 ± 0.17	1.16 ± 0.16	1.05 ± 0.03	1.16 ± 0.07
Triacylglycerols (mg/g)	0.14 ± 0.02	0.27 ± 0.08	0.12 ± 0.02	0.12 ± 0.01
Lactate (mg/g)	0.011 ± 0.005	0.014 ± 0.004	0.013 ± 0.006	0.009 ± 0.002
Glycogen (mg/g)	0.27 ± 0.11 ^a^	0.79 ± 0.09 ^b^	0.20 ± 0.07 ^a^	0.23 ± 0.06 ^a^

**Table 3:** Skeletal muscle glucose, triacylglycerols, lactate and glycogen content in vehicle (sham-control), cortisol, BSA and cortisol-BSA administered fish at 72 h post-treatment. Results are expressed as mean ± SEM (*n* = 8). Different letters indicate significant differences between groups (*p* value < 0.05). All data were analyzed using one-way ANOVA and Tukey’s HSD as a post hoc test.

Parameter	SHAM	CORT	BSA	CORT-BSA
Glucose (mg/g)	2.16 ± 0.14	2.18 ± 0.08	1.94 ± 0.03	2.05 ± 0.09
Triacylglycerols (mg/g)	1.29 ± 0.54	2.94 ± 1.17	0.45 ± 0.15	1.17 ± 0.34
Lactate (mg/g)	0.29 ± 0.02	0.34 ± 0.03	0.24 ± 0.02	0.27 ± 0.02
Glycogen (mg/g)	0.66 ± 0.08	0.97 ± 0.12	0.33 ± 0.06	0.79 ± 0.15

## Data Availability

The commercial reagents used in this study were listed in the Section 2. The nucleotide sequences used in this study were collected from the National Center for Biotechnology Information (NCBI) GenBank repository and from the available database belonging to the *S. aurata* sequencing project [23].

## Supplementary Materials

The following are available online at https://www.mdpi.com/article/10.3390/ani11113310/s1, Table S1: List of primers used for RT-qPCR. Eff % means efficiency percentage, and TM means melting temperature, Figure S1: Correlation between both gluconeogenesis-related genes expression, *pepck* (A) and *g6pc* (B), and plasma glucose levels for each fish.
