# Supplementary material for: Differential Metabolic and Transcriptional Responses of Gilthead Seabream (Sparus aurata) Administered with Cortisol or Cortisol-BSA
Source: Animals (Basel). 2021 Nov 19;11(11):3310. doi: 10.3390/ani11113310 (PMC8614361; doi:10.3390/ani11113310)
Supplement: Supplementary file 1 [file animals-11-03310-s001.zip › Figure S1.pdf]

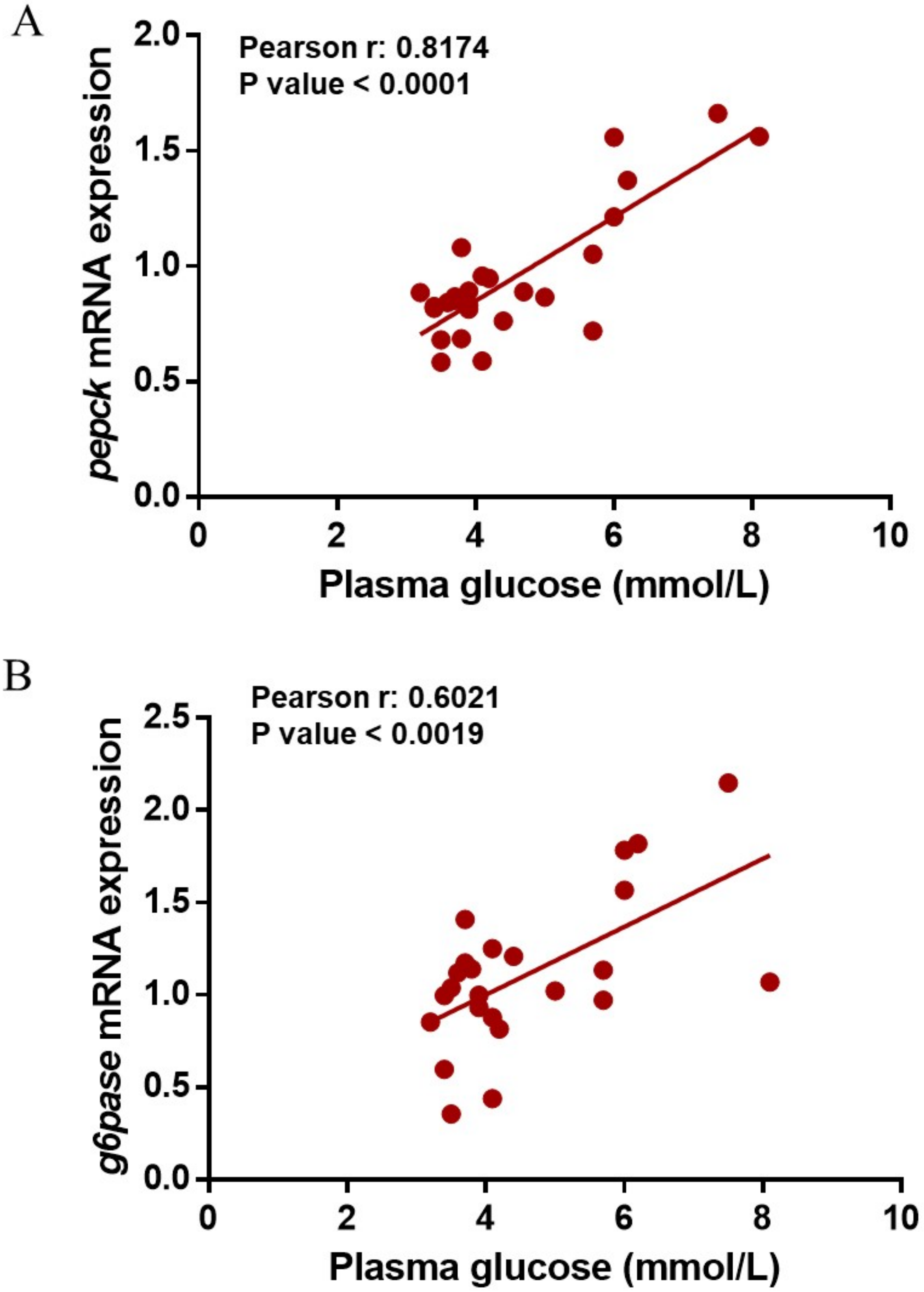

Figure S1. Correlation between both gluconeogenesis-related genes expression, *pepck* (**A**) and *g6pc* (**B**), and plasma glucose levels for each fish.
